# An Update on Corneal Biomechanics and Architecture in Diabetes

**DOI:** 10.1155/2019/7645352

**Published:** 2019-06-02

**Authors:** María A. del Buey, Paula Casas, Constanza Caramello, Nuria López, Marisa de la Rica, Ana B. Subirón, Elena Lanchares, Valentín Huerva, Andrzej Grzybowski, Francisco J. Ascaso

**Affiliations:** ^1^Department of Ophthalmology, Hospital Clínico Universitario “Lozano Blesa”, Zaragoza, Spain; ^2^Aragón Health Research Institute (IIS Aragon), Zaragoza, Spain; ^3^Aragon Institute of Engineering Research (13A), University of Zaragoza, Zaragoza, Spain; ^4^University of Zaragoza, Zaragoza, Spain; ^5^Centro de Investigación Biomédica en Red en Bioingeniería, Biomateriales y Nanomedicina, (CIBER-BBN), Barcelona, Spain; ^6^Department of Ophthalmology, Hospital Universitario Arnau de Vilanova, Lleida, Spain; ^7^Department of Ophthalmology, University of Warmia and Mazury, Olsztyn, Poland; ^8^Institute for Research in Ophthalmology, Foundation for Ophthalmology Development, Poznan, Poland

## Abstract

In the last decade, we have witnessed substantial progress in our understanding of corneal biomechanics and architecture. It is well known that diabetes is a systemic metabolic disease that causes chronic progressive damage in the main organs of the human body, including the eyeball. Although the main and most widely recognized ocular effect of diabetes is on the retina, the structure of the cornea (the outermost and transparent tissue of the eye) can also be affected by the poor glycemic control characterizing diabetes. The different corneal structures (epithelium, stroma, and endothelium) are affected by specific complications of diabetes. The development of new noninvasive diagnostic technologies has provided a better understanding of corneal tissue modifications. The objective of this review is to describe the advances in the knowledge of the corneal alterations that diabetes can induce.

## 1. Introduction

The first World Health Organization (WHO) global report on diabetes mellitus indicates that the number of adults living with this disorder has almost quadrupled since 1980 to 422 million adults. This large increase is due mainly to a higher incidence of type 2 diabetes (T2D) and the influence of factors such as overweight and obesity [1]. Diabetes is a systemic metabolic disease associated with high morbidity and mortality that can affect almost all tissues of the human body, including the most superficial and transparent ocular tissue: the cornea [2–7]. The prolonged high blood glucose levels that occur in diabetes can cause severe ophthalmological complications that affect both the anterior and posterior segments of the eye and can produce a significant visual deficit, including blindness. The eyeball is an organ accessible to noninvasive exploration and can provide great information about the possible involvement of other systemic organs caused by diabetes. The different corneal components (epithelium, stroma, nerves, and endothelium) are each affected by specific complications related to diabetes and poor glycemic control. It is well known that diabetic retinopathy is a good indicator of the state of microvascular disease in the rest of the organs. In the same way, the changes in corneal structures that we can recognize with new noninvasive technologies could predict systemic complications of diabetes or evaluate control of the disease. These changes in the corneal nerves of patients with diabetes could predict systemic conditions such as peripheral and autonomic neuropathy, while the state of the endothelial cells or changes in corneal thickness could inform on the status and level of control of the disease. The possibility of identification of structural and biomechanical changes of the cornea in patients with diabetes by means of accessible and noninvasive techniques can offer a new possibility for the early treatment of possible systemic complications. An improved knowledge of the changes produced by diabetes in the cornea and advances in diagnostic technology made in the last 10 years have led to substantial progress in our understanding of the biomechanics and architecture of the cornea. This review summarizes advances in our knowledge of the clinical manifestations and the “layer by layer” corneal changes that diabetes can produce.

## 2. Materials and Methods

We have carried out a systematic review of the literature published between January 1, 2008 and November 1, 2018 concerning the role of diabetes in structural and biomechanical changes in the cornea. A literature search was conducted in the NCBI Entrez PubMed database combining the term “diabetes” with a series of key words such as “corneal epithelium,” “corneal thickness,” “corneal stroma,” “corneal biomechanics,” “ocular response analyzer,” “corneal hysteresis,” “corneal nerves,” and “corneal endothelium.” Of the 314 manuscripts registered initially, those that were duplicated or without a summary in English were excluded, and 243 articles were finally examined by the coauthors to determine their relevance. The articles that included only the posterior segment were considered not relevant. A total of 81 papers were deemed irrelevant.

## 3. Diabetes and the Corneal Epithelium

Diabetes is associated with ocular surface disorders such as dry eye, superficial punctate keratitis, recurrent corneal erosion syndrome, and persistent epithelial defects [8, 9]. The underlying and responsible mechanisms that have been suggested for the appearance of these pathologies are a loss of corneal innervation (see Corneal Nerves in Diabetes), loss of basal epithelial cells, production and accumulation of advanced glycation end products (AGEs), disruption of tight junctions between epithelial cells, and disruption of trophic factors that encourage wound healing.

### 3.1. Basal Epithelial Cell Density (BECD)

Cai et al. [10] evaluated the effects of type 1 diabetes (T1D) on the whole cornea, corneal sublayer thickness, and basal epithelial cell density (BECD) using in vivo corneal confocal microscopy (CCM) in a streptozotocin-induced diabetic mouse model. They found reduced BECD and a decreased thickness of the corneal epithelium in these diabetic mice. Dehghani et al. [11] reported a decrease in the thickness of basal and intermediate epithelial cell density in a human in vivo case-control study with laser-scanning CCM in a cohort of diabetic patients. Similar results were obtained by Szalai et al. [12] and Qu et al. [13], who also found a significant decrease in the cell population of the basal epithelial layer. Different mechanisms have been proposed as causal for this outcome, including decreased innervation at the subbasal nerve plexus (SBNP) (see Corneal Nerves in Diabetes), increased basement membrane thickness, or metabolic dysfunctions associated with the accumulation of AGEs in the basal membrane [11, 14].

### 3.2. Epithelial Basement Membrane

Classically, diabetes has been associated with corneal epithelial basement membrane (BM) disorders [15–17]. BM becomes irregularly thickened and multilaminated, with abnormal adhesions to the supralying epithelium [18], and has been related to accumulation of AGEs. This enlarged configuration of the basal membrane leads to subclinical scattering of light in the cornea visible on in vivo CCM, but not detectable on routine clinical examination [19, 20]. Recently, Özyol and Özyol [21], by using Scheimpflug tomography in a cohort of diabetic patients scanned by densitometry, detected that the anterior corneal layer displayed significantly higher values on light scattering in diabetic eyes than in the eyes of controls.

Regarding the biochemical changes in the composition of the corneal BM, Ljubimov et al. [17, 22] reported a markedly diminished change with a weak staining for chains of laminin-l, entactin/nidogen, laminin-10, and a3-a4 chains of type IV collagen in diabetic corneas with diabetic retinopathy. Saghizadeh et al. [23] also found reduced immunostaining of laminins, entactin/nidogen-1, and laminin receptor integrin *α*3*β*1. In addition, they report a significant decrease in the laminin *γ*3 chain and fibronectin [24]. Different hypotheses could be responsible for these changes in the composition of the corneal BM, an increase in the activity of the proteinases, and a decrease of growth factors or diffusion from the vitreous or the retina of pathological substances associated with hyperglycemia may vary the composition of the corneal BM. Moreover, it has been suggested that changes in the composition of the corneal BM in diabetic patients could alter the interaction between epithelial cells and the underlying basal membrane, triggering variations in the expression patterns of integrins [25].

### 3.3. Tight Junctions

The major function of the corneal epithelium is to protect the interior of the eye; the corneal epithelium creates “tight junctions”—physical and chemical barriers that protect against infection, maintaining corneal transparency and integrity. Epithelial cell junctions, visualized as electron dense structures, play an important role in the formation and maintenance of the epithelial barrier, homeostasis, and host defense of the cornea.

Huang et al. [26], using a diabetes rat model, found delayed corneal healing with fewer multilayers of epithelium covering the denuded surface at 48–72 hours, with increased disorganization of occludin protein stained with immunofluorescence. Scanning electron microscopy revealed abnormal intercellular connections, fissures between cells, a decrease in the number of microvilli, and dropsy in the diabetic rat group. Yin et al. [27] reinforced this idea when they observed a delayed, but not absent, formation of tight junctions between cells during the healing process of epithelial corneal ulcers in diabetic rats. There are no studies in humans that corroborate these findings in animal models.

### 3.4. Advanced Glycation End Products (AGEs)

AGEs have been proposed as the cause of the abnormalities seen in the cornea of patients with diabetes. They are a heterogeneous group of substances that result from the nonenzymatic glycation and oxidation of proteins and lipids. AGEs stimulate cell apoptosis by increasing intracellular reactive oxygen species (ROS) production [28, 29].

The accumulation of AGEs leads to alterations in tissue function. AGE accumulation has been detected at the site of the corneal epithelium and epithelial BM in diabetic rats [30, 31] and monkeys [32] and in human diabetes patients [29]. In addition, it has been shown that the AGE concentration is elevated in the tears of human diabetes patients [33]. Kim et al. [30] demonstrated both the accumulation of AGEs and the presence of oxidative DNA damage in diabetic corneal cells. They found a correlation between the apoptotic damage in the diabetic cornea and the intense nuclear localization of a marker of oxidative DNA damage (8-hydroxydeoxyguanosine). These findings provide strong evidence that nuclear oxidative DNA damage by AGE accumulation is responsible, at least in part, for the apoptotic damage of diabetic corneal cells, leading to delayed epithelial wound healing in the diabetic cornea.

### 3.5. Wound Healing

Several authors have recently demonstrated delayed wound healing in diabetic rat models [27, 34, 35]. Longer healing times than those in the control group were observed in a group of diabetic rats in which a mechanical debridement had been performed. Growth factors and cytokines are powerful regulators of cell behavior and promote tissue wound healing. Disruption of trophic factors has been identified as being responsible for delayed corneal healing in both human and animal models of diabetes. An important example is epidermal growth factor receptor (EGFR); this pathway is critical for cell migration and proliferation and is a major mediator of corneal epithelial wound healing [36]. Several authors have reported disruption of this pathway in the cornea of diabetic rats [27] and in human corneal epithelial cells [37, 38].

Another altered pathway is mediated by hepatocyte growth factor (HGF) which is involved in the processes of cellular proliferation, migration, and apoptosis [24, 39] The HCG receptor, the proto-oncogene c-Met, is apparently involved in activation of p38 mitogen-activated protein kinase (p38 MAPK) which has been related to stimulation of corneal epithelial migration [40]. Saghizadeh et al. reported an increased expression of HGF and a diminished c-Met expression in the diabetic cornea [41]. Recent studies carried out by the same group of researchers have developed an adenoviral-based gene therapy in human diabetic cultured corneas, improving wound healing times by normalizing the levels of c-Met expression, associated or not with the normalization of other proteinases or kinases whose values are usually altered in the corneas of diabetic patients [24, 41–44].

Other routes which have recently been studied include Serpine 1 [35], which, when compared to controls, is significantly diminished in corneal epithelium collected from diabetic rats. In addition, opioid growth factor (OGF) [45, 46], which is elevated in the plasma of patients with diabetes, acts as a negative regulator of epithelial proliferation and wound healing. When OGF joins to its specific receptor, OGFr, they are able to inhibit cell replication [46]. Moreover, it has been observed that opioids antagonists such as naltrexone, which block the axis OGF-OGFr, favor cell replication and therefore tissue remodeling [45].

Likewise, insulin-like growth factor sun-1 (IGF-1) and its receptor, which are found in human corneal keratocytes and epithelial cells, mediate cell migration, proliferation, and survival. It appears that elevated levels of insulin-like growth factor binding protein 3 (IGFBP-3) found in the tears of diabetic human subjects may attenuate IGF-1 receptor signaling in the diabetic cornea [47]. According to Wang et al. [48,49], this attenuation via IGFBP3/IGF-1 could be promoted by Sirtuin 1 (silent mating type information regulation 2 homolog), a protein that belongs to the group of class III histone/protein deacetylases. In addition, Shen et al. [50] reported that corneal wounds in diabetes have abnormal electric signals which may contribute to impaired wound healing, possibly via cell electrotactic migration disruption, and even suggest electrical stimulation as a new therapeutic option in the management of chronic and nonhealing wounds.

## 4. Diabetes and Corneal Stroma

### 4.1. Corneal Nerves in Diabetes

The structure of the corneal nerves is very important in maintaining a healthy ocular surface. The cornea is the most densely innervated tissue in the human body (approximately 7,000 nociceptors per mm^2^) [51]. This great sensitivity serves to protect the cornea. The corneal nerves are derived from the ciliary nerves that form the terminal branches of the ophthalmic division of the 5th cranial nerve. These bundles of nerves penetrate radially in the middle and anterior corneal stroma through the limbus and then bifurcate and advance towards the epithelium as long bundles, fine branches, and nerve terminals [52]. This results in a moderately dense midstromal plexus and a dense subepithelial plexus, whose branches cross Bowman's membrane to form an SBNP complex that emits nerve terminals that innervate all epithelial layers [53]. The different types of nerve endings (nociceptive, temperature, and polymodal) are responsible for sensations such as pain, touch, temperature, and dryness, which are very important for the reflex of blinking, the production of tears, and the healing of lesions [54–58].

Diabetes is a systemic condition that can affect corneal innervation and sensitivity, causing complications that can lead to blindness. Patients with diabetes show a reduction in corneal sensitivity, clinically measured with an esthesiometer [59], due to a progressive decrease in the density of the corneal nerves [60]. Advances in technology have allowed for rapid, noninvasive, and high-quality visualization of the corneal structure using in vivo CCM. The corneas of patients with diabetes show a lower density of SBNP, a reduction of epithelial nerve fiber bundles per image with decreased branches, and greater nervous tortuosity than the corneas of healthy patients [61, 62]. These alterations are associated with a reduction in corneal sensitivity in patients with diabetes [63]. He and Bazan studied the architecture of corneas donated by patients with insulin-dependent diabetes of varying duration. Although they did not find differences in the number of nerve trunks of the stroma, they found a decrease in the density of epithelial nerves in the corneas of patients with 5 or more years' duration of insulin-dependent diabetes. The presence of abundant loops of nerve fibers in the corneal stroma, which appeared to be formed as a result of resistance in the BM to the penetration of the stromal nerve branches in the epithelia, was also observed [64].

Damage to the corneal nerve fibers leads to an alteration of the healing process of the wounds and greater susceptibility to infections; this damage causes most of the symptoms experienced by diabetes patients with keratopathy, such as decreased corneal sensitivity, recurrent corneal erosions, persistent epithelial defects, and neurotrophic corneal ulcers [65–67].

Examination of the corneal nerves and exploration of corneal sensitivity are useful tools for the early detection and evaluation of peripheral neuropathy in patients with diabetes. Several studies have shown that CCM is a valid, accurate, noninvasive method to identify small nerve fiber pathology; CCM can also be used to diagnose diabetic neuropathy [68, 69]. It has been found that corneal nerve fiber density and length, as well as corneal nerve branch density, are significantly reduced in patients with diabetic polyneuropathy when compared to control subjects. The diagnostic efficiency of CCM is comparable to intraepidermal nerve fiber density by skin biopsy; however, CCM may be preferred due to its rapid, automated, and noninvasive characteristics [69]. What is important to recognize is that CCM can identify nerve alterations in the cornea that precede the clinical signs and symptoms of peripheral neuropathy, nephropathy, or diabetic retinopathy. Asghar et al. observed alterations even in patients with impaired glucose tolerance but who did not meet the clinical criteria of T2D [70]. CCM is also useful in the assessment of a patient's response to treatments, since it has been found that there is a recovery of corneal SBNP and an improvement of neuropathy in diabetes patients who have received a double pancreas and kidney transplant [71, 72]. A recent study using in vivo CCM has found that nerve fiber damage in T1D correlates with the degree of diabetic retinopathy. Furthermore, studies show that T1D patients with higher age at diagnosis have a higher nerve fiber density. These results indicate that age at T1D diagnosis potentially has an important effect on final nerve fiber density [73]. In conclusion, studies show that CCM offers an early, faster, and less invasive diagnosis of diabetic peripheral neuropathy than current gold standard techniques such as nerve electrophysiology, sural nerve biopsy, and skin puncture biopsy.

### 4.2. Corneal Stroma Structure and Biomechanics in Diabetes

The stroma represents 90% of the corneal thickness; its special structure and composition give the cornea its biomechanical properties [74]. The highly differentiated ultrastructure of the corneal stroma, with its special orientation, diameter, and separation of fibrillar collagen bundles and the regulatory role of other components of the extracellular matrix (proteoglycans and glycosaminoglycans), confer transparency and biomechanical behavior to the cornea [75, 76]. The way in which diabetes affects the structure and function of the corneal stroma is not well known; there have been numerous studies in recent years into how diabetes affects corneal thickness and the biomechanical properties of the corneal stroma. The main points of interest in the reviewed papers on corneal biomechanics in diabetes involved the in vivo measurement of the corneal biomechanical properties; this was largely due to the recent development of technological devices to quantify some of these properties. The first of these was the Ocular Response Analyzer (ORA, Reichert Ophthalmic Instruments, Depew, NY, USA), and more recently the Corvis ST (Corvis ST; Oculus, Wetzlar, Germany). In addition, in the last two years, details of corneal optical densitometry (COD) analysis using the Pentacam HR imaging system in diabetes patients have been published.

#### 4.2.1. Corneal Thickness

Recently published research findings on corneal morphology show evidence of greater central corneal thickness (CCT) in patients with T2D [77–80]. In studies of corneal thickness in patients with diabetic retinopathy, no statistical differences were found between groups of patients with proliferative retinopathy or nonproliferative retinopathy and those without diabetic retinopathy [81–85]. These results indicate that diabetes patients have a significantly thicker CCT, regardless of the state of retinopathy. Santiagu et al. [86] found that diabetes during pregnancy also does not seem to influence CCT. In a recent article, Kumar et al. [87] showed that CCT increases in relation to the severity of peripheral diabetic neuropathy due to an increase in stromal thickness. Other studies, however, have not found an increase in CCT in cases of T1D [88] or T2D patients [89–91]. Similarly, studies of patients with primary open-angle glaucoma (POAG) did not show differences in CCT between groups of glaucoma patients with and without diabetes [92, 93]. Hashemi et al. [94], in a five-year follow-up study, showed that overall patterns of change in CCT and corneal shape in diabetes patients over 40 years of age were similar to those observed in those individuals without diabetes. However, changes related to age in the thickness, volume, and shape of the central and peripheral cornea were less pronounced in subjects with diabetes.

Several studies on corneal thickness and biomechanics have been conducted in children with T1D. Tiutiuca [95] conducted a study in 100 children with T1D in Romania that showed an increase in CCT when compared to an equivalent number of healthy children. These results are comparable to those from a similar study conducted in Turkey by Akinci et al. [96]. However, other studies have not found this increase in CCT in children or young people with T1D [97, 98]. In another Turkish study in children with T1D, CCT was not shown to be associated with either the current fasting glucose level or duration of disease [99]. However, in a recent clinical paper on corneal thickness in T1D, higher CCT values were observed in acute hyperglycemia state, when compared with those obtained after 48 hours of metabolic compensation, concluding that corneal pachymetry can potentially serve as a promising method for noninvasive evaluation of the increased risk of developing cerebral edema in patients with T1D [100].

#### 4.2.2. Biomechanical Properties

ORA and Corvis ST are noncontact devices that provide tonometry and corneal displacement measurements via the injection of a rapid jet of air. ORA was the first device capable of evaluating in vivo biomechanical properties such as corneal hysteresis (CH) and corneal resistance factor (CRF), calculated from the differences in pressures that act to achieve defined corneal deformation states. In addition, ORA provides the intraocular pressure (IOP) correlated with the Goldmann IOP (IOPg) and the compensated corneal IOP (IOPcc). CH predominantly reflects the viscous properties of corneal tissue, whereas CRF is an empirically derived measurement representative of the elastic properties of the cornea [101]. Both parameters are derived from a complex interaction between the collagen composition of the cornea, its thickness, hydration, age, and other physiological factors [102, 103]. Studies have shown that lower CH values may be associated with several disorders such as keratoconus, Fuchs endothelial corneal dystrophy, and glaucoma [104–106]. The measures provided by the ORA have not been affected by CCT values [107].

Table 1 summarizes the publications in the last ten years that concern biomechanical corneal properties measured with ORA in diabetes patients. In most of the cross-sectional studies reviewed, it has been found that subjects with diabetes have higher CH values than the population without diabetes [74, 91, 92, 108, 110, 112, 115–118]. Only three studies [109, 113, 114] reported that subjects with diabetes have a lower CH when compared to age-matched controls, and four others did not find significant differences in CH values between populations with and without diabetes [99, 111, 115, 119]. A possible relationship between increased CH and the control of diabetes has also been investigated. Kotecha et al. [110] found that the level of glucose in the blood correlated significantly (but weakly; *r* = 0.28) with Hashemi et al. [94] found that subjects with fasting blood glucose values greater than or equal to 7.0 mM had higher CH and CRF values than those with glucose values less than 6.1 mM. Regarding corneal biomechanical properties in diabetic children, two studies show that T1D does not have any effect on corneal biomechanical parameters (CH and CRF) in childhood [99, 111] (Table 1). We found only one study that analyzed the results of these biomechanical parameters measured with Corvis ST in a diabetes population: Perez-Rico et al. [113] found differences in some parameters of corneal deformation in the diabetic population, with an increase in the time of the first applanation and a significant decrease in some parameters, such as the time of second applanation, the velocity of the first applanation, and the maximum deformation amplitude at the corneal apex.

#### 4.2.3. Intraocular Pressure (IOP)

POAG patients, both with and without diabetes have also been studied using ORA. In a study by Castro et al. [92], in which 74 eyes of 44 POAG patients were evaluated, it was found that CH was significantly higher in POAG patients with diabetes compared to POAG individuals without diabetes, without finding differences in the CCT. CRF, diabetes duration, and the effect of metabolic control on corneal biomechanical properties were not evaluated in this study. More recently, Akkaya et al. [93], in a study of 101 eyes of 101 patients, found that CH in diabetes was similar, but CRF, mean rim area, and rim volume (measured by optical coherence tomography) were found to be significantly higher in POAG patients with diabetes when compared to POAG patients without diabetes (Table 1). The results of these studies could suggest a protective role of diabetes in patients with glaucoma.

Several studies indicate a relationship between diabetes and higher IOP values [78, 109, 112–114], but this association is controversial. On one hand, diabetes is associated with a thicker CCT, but a thick cornea also provides higher IOP values. Luo et al. [120], in an extensive study, assessed both the direct and indirect effect of diabetes on IOP through the CCT mediator. They found that diabetes was associated with higher IOP, and CCT only contributed in a small proportion to the total effect of diabetes on IOP. This direct association between diabetes and IOP may have a pathophysiological importance with respect to the risk of glaucoma in people with diabetes.

#### 4.2.4. Corneal Densitometry

Some studies on COD analysis using the Pentacam HR imaging system in diabetes have been recently published. COD is used to describe the characteristics of the corneal tissue and makes it possible to quantify its degree of transparency. Previous findings showed that COD in an area of inflammation was higher than normal, even when the damage was repaired [121]. It has also been confirmed that Pentacam HR objectively assesses a nubecula through a quantitative measurement of corneal density [122]. Gao et al. [123] used Pentacam HRto assess CCT, COD, and alterations of corneal transparency in 180 diabetes patients; they found an increase in COD and CCT compared with controls, with a positive association between the medial and intimal COD and central CCT in diabetes patients. In addition, Calvo-Maroto et al. [124], in a pilot study in adult diabetes patients, showed higher values of corneal light backscatter in patients with diabetes when compared with healthy subjects. However, COD values in children with T1D were similar in all concentric zones and layers to those in healthy children [125]. These findings suggest that there is an influence of the age and/or time of evolution of diabetes in the degree of corneal transparency or COD as determined by Pentacam HR.

#### 4.2.5. Analysis of Findings

The reason why diabetes is associated with increased CCT in cases without corneal epitheliopathy is still unknown. It has been speculated that there may be an accumulation of AGEs in the corneal stroma of patients with diabetes, along with a nonenzymatic cross-linking between the collagen fibers and the proteoglycans. This cross-linking could theoretically explain the greater rigidity and thickening of the cornea in diabetics (higher CH, CRF, and CCT in some studies). Zou et al. [32] compared eight monkeys with insulin-dependent diabetes (induced by streptozotocin injection) with four controls, and found a cross-linking with abnormal aggregates of collagen fibrils in the stromal matrix on transmission electron microscope examination in monkeys with diabetes. In another recent experimental study in rabbits, Bao et al. [126] investigated the effects of diabetes on the behavior of the cornea, showing a significant increase in AGEs, CCT, and IOP in rabbits with diabetes. In addition, the tangent modulus of the cornea at four stress levels was significantly higher in rabbits with diabetes, indicated by greater mechanical rigidity of the cornea. These findings are consistent with evidence presented by Goldin et al. [127] in relation to the AGE-induced cross-linking of the extracellular matrix of certain tissues in patients with diabetes, which results in an increase in arterial stiffness. The fact that children with diabetes have the highest CCT without evidence of other systemic complications of diabetes suggests that AGEs may affect the cornea before other organs [95, 104] and that a test as accessible as pachymetry may be used to detect early changes.

The determination of corneal biomechanical properties can provide information on changes in the extracellular matrix in the eyes of diabetes patients and could therefore offer a new parameter for monitoring the state of the disease. In this review, we have found several studies conducted with ORA that have investigated the influence of diabetes on the biomechanical parameters of the cornea, but with somewhat contradictory results. Most of them (Table 1) find higher CH values in diabetes patients that could be caused by changes in the fundamental substance of the cornea, which would modify its viscosity [74, 108, 113, 115]. The oxidative stress caused by sustained hyperglycemia leads to the formation of AGEs (by nonenzymatic glycosylation) that accumulate in the tissues; in addition, a glycation of proteoglycans and glycosaminoglycans of the matrix is proposed, which would modify the viscosity of the cornea, increasing the CH [74, 115].

In addition, there are further pathogenic factors that could modify the biomechanical properties of the cornea in diabetes patients; these should be considered to clarify some contradictory results in the published evidence. A dysfunction of the epithelial and endothelial cells of the cornea could alter control of hydration of the cornea, causing subclinical edema that could influence the results by causing a decrease in CH and CRF, as well as an increase in CCT [74, 105, 126]. This hypothesis could explain the decreased CH values reported in some studies [109, 113, 114] and the elevated CCT in most of the studies [77–80]. Factors such as axial length [128], possible endothelial dystrophy [105], the existence of a subclinical keratoconus [129], or lubrication of the surface [130] can produce significant biomechanical changes that should be considered in future studies. In addition, to determine how the parameters would change during progression of the disease, measurement of the biomechanical properties in the same patients over time would be necessary. In future, we expect interesting findings regarding the biomechanical properties of the cornea in diabetes.

## 5. Diabetes and Endothelium

Table 2 summarizes the publications in the last ten years that concern endothelial status in diabetes patients, compared in most cases with healthy controls.

The italicized publications in Table 2 did not find statistically significant disagreement between the endothelial cell density (ECD) of diabetes patients when compared with healthy controls [72, 134, 135]. However, the majority of authors found differences in the endothelial cell population in individuals with versus without diabetes; the number of cells is decreased in diabetes patients, especially in those with T1D [12, 132, 133, 136]. Calvo-Maroto et al. [139] studied the effect of diabetes duration and poor glycemic control on the endothelial cell population: they found that the longer the evolution time of diabetes, the greater the loss of endothelial cells; this could be the reason why we find more differences in T1D patients, who are generally of a younger age at disease onset and usually present a longer duration of diabetes evolution. Islam et al. [138], Anbar et al. [136], and Urban et al. [133] also found this correlation between diabetes duration and ECD.

According to Storr-Paulsen et al. [134], and although they did not find statistically significant differences between groups with respect to ECD, higher glycated hemoglobin A1C levels were associated with lower ECD. Similar findings were described by Módis et al. [132] in T1D patients. Therefore, we can conclude that patients with longer disease evolution times and with poor metabolic control are those with higher endothelial loss.

Regarding endothelial characteristics, diabetes patients seem to have higher rates of polymegathism and lower percentages of hexagonality (higher polymorphism) [79, 131, 136, 137]. Moreover, Anbar et al. [136] and Islam et al. [138] found a significant correlation between the duration of diabetes and pleomorphism and polymegathism, supporting the idea that the longer the disease evolution, the more the endothelial alteration.

Another indicator of endothelial cell dysfunction, along with ECD, pleomorphism, and polymorphism, is CCT. The healthy cornea stays in a state of dehydration, as endothelial cell Na^+^/K^+^ ATPase and tight junctions are responsible for limiting the entrance of aqueous humor into the stroma [140]. When there is a substantial endothelial loss, the decrease in the number of tight junctions between cells allows more fluid to enter the stroma, favoring stromal rehydration with increased CCT that can lead to a loss of corneal transparency. Several authors have reported higher CCT in T1D [12, 133, 136] and T2D [77, 134] patients compared to controls, and Calvo-Maroto et al. [139] reported higher CCT in long-term T2D patients (diagnosed and treated for ten years or more) when compared with short-term T2D patients and controls.

Endothelial changes in the diabetic cornea can alter their function. Abnormal morphology of the corneal endothelial cells combined with increased CCT is an indicator of alterations of endothelial pump function, which can lead the cornea to a greater risk of decompensation following surgical trauma. Thus, a complete endothelial examination is important before ophthalmological procedures such as cataract surgery, since it is associated with an endothelial loss [141, 142].

### 5.1. In Vitro Studies

In vitro studies carried out over the last ten years with respect to the effect of diabetes on the corneal endothelium are summarized in Table 3. The findings in these donor tissue banks studies support the data observed in in vivo studies. Chocron et al. [148] and Liaboe et al. [145] reported lower levels of ECD in diabetes patients when compared to controls. Chen et al. [147] described this endothelial loss only in patients between 21 and 60 years; subjects above this age did not have statistically significant differences when compared to healthy controls. Moreover, Kwon et al. [143] report that age, previous cataract surgery, and diabetes were found to be the most important risk factors for deficient donor quality with respect to ECD.

Schwarz et al. [144] designed a method to assess differences in endothelium/Descemet membrane complex adhesion strength from stroma between diabetic and nondiabetic donor corneas. They did not find differences in ECD, hexagonality, or coefficient of variation of cell area between diabetes patients and controls; nevertheless, they observed greater resistance in diabetes patients for the separation between the endothelium/Descemet complex and the stroma.

There are two publications that analyze mitochondrial functioning in the endothelium of diabetes patients. Aldrich et al. [146] report that endothelial cells from insulin-dependent diabetes patients with medical complications had variations in their mitochondrial configuration, notable Golgi bodies associated with numerous vesicles, collection of lysosomal bodies/autophagosomes, and focal production of abnormal long-spacing collagen. Skeie et al. [149] found a decrease in mitochondrial proteins in corneas taken from patients with insulin-dependent diabetes when compared to those from patients with non-insulin-dependent diabetes. They suggest that proteins implicated in mitochondrial dysfunction decrease to a greater extent as diabetes progresses to insulin dependence, indicating that mitochondrial changes may be linked to diabetes insulin therapy itself or disease conditions at the time of transition to insulin therapy.

## 6. New Therapeutic Perspectives

In the past decade, certain therapies to treat specific corneal disorders in diabetes patients have been investigated. On one hand, these patients can benefit from the available symptomatic treatment options, such as artificial tear eye drops, topical anti-inflammatory drugs [150] (NSAIDs, steroids, and cyclosporine A), contact lenses [9], autologous serum, or platelet-rich plasma [4, 151]. It is also known that a strict metabolic control of blood glucose levels is important for prevention and treatment of ocular surface alterations in patients with diabetes [9]. On the other hand, new specific therapies for diabetic keratopathy and neuropathy are being investigated, even though they are in an experimental phase. Local therapy with substance P and IGF-1 has been shown to be effective in the treatment of diabetic keratopathy [152, 153], but more studies are needed to determine its effects on other ocular structures before its use can be recommended. There have also been studies that assess the effectiveness of substances such as aldose reductase inhibitor [154], the anti-inflammatory and healing agent TB4 [155], topical NGF [156], resolvin D [157], oral nicergoline [158], and antioxidants such as carnosine and *β*-carotene [159]. However, most of the suggested therapies have been investigated in animal models. A promising agent that has shown efficacy in several animal studies is naltrexone, an opioid antagonist which blocks opioid-receptor binding, thereby accelerating DNA synthesis [9]. In diabetes, there is an inhibition of cell proliferation due to the production of excessive opioid growth factors. The topical application of naltrexone has been shown to be useful both for corneal regeneration and tears production, improving the corneal sensitivity in T1 and T2 diabetic animal models [45, 160]. In addition, there are promising novel therapeutic approaches that include gene [23, 24, 41] and stem cells therapies [4, 44]; nevertheless, at the moment, they are in preclinical development. In the near future, we can expect some advances in the prevention and management of corneal disorders associated with diabetes, possibly from a multidisciplinary point of view.

In conclusion, different corneal components (epithelium, stroma, nerves, and endothelium) suffer specific complications of diabetes. The development of new noninvasive diagnostic technologies has provided a better understanding of corneal tissue changes related to diabetes. The published literature sheds light on the potential utility of the biomechanical corneal properties to improve our understanding of the mechanical behavior of this complex tissue in diabetes patients. However, the literature shows controversial results in relevant areas such as CH and its impact on IOP measurement. New technologies are showing promise in consolidating the utility of the biomechanical corneal properties as a clinical tool and a relevant field for the future improvement of diagnosis of diabetes and control of the disease.

## Figures and Tables

**Table 1:** Summary of prospective cross-sectional studies of CH, CRF, IOPg, and IOPcc in diabetes patients.

Author, year, country	Study groups/sample size	Mean age (years)	ORA parameters (mean mmHg) controls/diabetes	Outcomes (*P* value)	Associations
Goldich, 2008, Israel [108]	40 with diabetes (40 eyes)/40 controls (40 eyes)	60.9/63.8	CH: 10.7 ± 1.6/9.3 ± 1.4	**0.0001**	(i) Subjects with diabetes had **higher CH and CRF** values than those without diabetes
			CRF: 10.9 ± 1.7/9.6 ± 1.6	**<0.0001**	
			IOPcc: 16.6 ± 4.4/17.7 ± 4.9	0.31	(ii) There was **no any** statistical **difference** between the groups in terms of **IOPg and IOPcc**.
			IOPg: 16.6 ± 4.3/16.1 ± 4.9	0.66	

Sahin, 2009, Turkey [109]	43 with diabetes (81 eyes)/61 control (120 eyes)	55.3/53.1	CH: 9.51 ± 1.82/10.41 ± 1.66	**0.0001**	**(i) CH** was found to be **significantly lower in diabetic** patients
			CRF: 10.32 ± 1.76/10.36 ± 1.97	0.8	(ii) There was no significant difference in terms of **CRF**
			IOPcc: 18.81 ± 4.71/15.85 ± 3.24	**0.0001**	**(iii) Mean CCT, GAT, IOPg, and IOPcc were significantly higher in diabetic** patients than in healthy control subjects
			IOPg: 17.68 ± 4.42/15.34 ± 3.66	**0.0001**	

Castro, 2010, Brazil [92]	44 **primary open-angle glaucoma patients**)		**CH:** 9.1 ± 1.9/7.8 ± 1.7	**0.04**	Diabetic patients presented significantly **higher CH** values than patients without diabetes. There was a significant and positive correlation between CH and CCT for all patients (*r* = 0.407, *P* < 0.001).
	19 with diabetes (34 eyes)/25 without diabetes (40 eyes				

Kotecha, 2010, UK [110]	61 with diabetes (61 eyes)	41.9/61.6/54.0	CH: 12.45 ± 1.74/10.90 ± 1.94/10.85 ± 1.68 CRF: 12.49 ± 2.01/11.50 ± 2.06/10.62 ± 1.64	0.008	(i) The **CH was significantly greater** in T1D patients.
	T1D (13 eyes)/T2D (48 eyes)/controls (123 eyes)			**0.0001**	(ii) The **CRF was significantly greater** in T1D and T2D patients.
					(iii) CH and CRF were weakly correlated with blood glucose concentration

Kara, 2012, Turkey [99]	46 T1D children (46 eyes)/50 controls (50 eyes)	14.2/14.5	CH: 12.3 ± 1.3/12.5 ± 1.5	0.609	**(i) CH and CRF in T1D are similar** to those of healthy controls.
			CRF: 12.4 ± 1.7/11.9 ± 1.5	0.152	(ii) IOPg and IOPcc in T1D are similar to those of healthy controls.
			IOPg: 17.4 ± 3.6/16.7 ± 2.9	0.232	
			IOPcc: 15.5 ± 3.4/15.1 ± 2.7	0.446	

Nalcacioglu-Yuksekkaya, 2014, Turkey [111]	68 T1D children (68 eyes)/74 controls (74 eyes)	12.7/12.9	CH: 10.8 ± 1.5/10.7 ± 1.7	0.624	**(i) CH and CRF in T1D are similar** to those of healthy controls.
			CRF: 10.9 ± 1.9/10.5 ± 1.6	0.207	(ii) IOPg and IOPcc in T1D are similar to those of healthy controls.
			IOPcc: 15.8 ± 3.0/15.3 ± 3.4	0.395	
			IOPg: 15.9 ± 3.7/15.2 ± 3.4	0.263	

Yazgan, 2014, Turkey [112]	156 with T2D (156 eyes)/74 controls (74 eyes)	57.75/57.91	CH: 10.37 ± 1.9/8.98 ± 1.4	**0.0001**	**CH, CRF, CCT, IOPg and IOPcc values were higher in diabetes** groups than controls. There was also a positive correlation between HbA1C level and intraocular pressure.
			CRF: 11.06 ± 2.3/8.99 ± 1.5	**0.0001**	
			IOPg: 17.63 ± 3.9/14.80 ± 2.9	**0.0001**	
			IOPcc: 17.70 ± 3.2/16.56 ± 2.4	**0.026**	

Pérez-Rico, 2015, Spain [113]	94 diabetic patients (94 eyes)	59.8/62.2	CH: 10.23 ± 1.83/10.9 ± 1.39/11.43 ± 1.69	**0.002**	(i) **CH** was significantly **lower** in diabetic patients with elevated HbA1c than in controls and was affected by disease duration, whereas the CRF remained unaltered.
	54 uncontrolled diabetes/40 controlled diabetes/41 controls		CRF: 11.05 ± 1.97/11.21 ± 1.97/10.53 ± 1.78	0.263	(ii) **IOPcc and IOPg were significantly higher** in diabetic patients with elevated HbA1c than in controls.
			IOPcc: 18.45 ± 3.79/14.68 ± 2.67/14.55 ± 3.72	**<0.0001**	
			IOPg: 18.16 ± 3.85/15.31 ± 3.14/14.46 ± 4.1	**<0.0001**	

Schweitzer, 2016, France [91]	Diabetes (137 eyes)/controls (695 eyes)	—	**CH:** 9.79/9.28	**0.003**	Subjects with diabetes had **higher CH and CRF values** than those without diabetes. Consistently, subjects having fasting blood glucose values greater than or equal than 7.0 mM had significantly higher CH and CRF mean values compared with subjects having fasting blood glucose values lower than 6.1 mM (*P* < 0.05).
			**CRF**:10.35/9.63	**0.003**	

Akkaya, 2016, Turkey [93]	**101 primary open-angle glaucoma patients (101 eyes)**		**CH**: 9.35 ± 1.49/8.86 ± 1.52	0.11	(i) **CH in diabetes was similar** to those of healthy controls.
	60 with diabetes (60 eyes)/41 without diabetes (41 eyes)		**CRF:** 10.15 ± 1.78/9.24 ± 1.92	**0.01**	(ii) RNFL thickness was measured by using Spectralis HRA + OCT.
					(iii) **CRF,** mean rim area, and rim volume were found to be significantly **higher** in the diabetic group when compared with nondiabetic group.

Bekmez, 2018, Turkey [114]	50 with T2D (50 eyes)/50 controls (50 eyes)	63.3/61.7	CH: 9.9 ± 1.5/10.5 ± 1.7	0.080	(i) There was **no any** statistical **difference** between the groups in terms of **CH and CRF**. However, mean CH and CRF values were found less in diabetic group.
			**CRF**: 10.4 ± 1.6/10.5 ± 1.7	0.730	
			IOPcc: 17.8 ± 3.6/16.0 ± 3.1	**0.006**	(ii) Corneal biomechanical differences seen in diabetic patients may be associated with significantly higher IOP measurements.
			IOPg: 16.9 ± 3.5/15.4 ± 2.9	**0.032**	

T1D = type 1 diabetes; T2D = type 2 diabetes; ORA = ocular response analyzer; CH = corneal hysteresis; CRF = corneal resistance factor; GAT = Goldmann applanation tonometry; IOP = intraocular pressure; CCT = central corneal thickness; IOPg = Goldmann-correlated intraocular pressure; IOPcc = corneal-compensated intraocular pressure.

**Table 2:** Summary of “in vivo” studies about endothelial status in diabetic patients compared with healthy controls.

Author, year	Type of study	Study groups	Technology	Parameters	Results
Shenoy, 2009 [131]	Case-control Prospective	110 diabetic patients (110 eyes) 27 T1D 83 T2D 110 controls (110 eyes)	NIDEK® confoscan 2.	ECD, coefficient of variability of cell size of cells showing polymegathism, percentage of hexagonal cells showing pleomorphism.	(i) ECD in eyes was negatively associated with the diabetes status. (ii) The coefficient of variability in endothelial cells with polymegathism was 12 (8 to 16) more among eyes of diabetic patients than that of controls. (iii) The corneal endothelial cells with pleomorphism were 9% less in controls compared to the diabetic subjects.
Módis, 2010 [132]	Case-control Prospective	21 insulin-dependent T1D patients (41 eyes) 30 patients with non-insulin-dependent T2D (59 eyes). Control group 1 (22 patients, 40 eyes). Age-matched normal subjects with T1D group) Control group 2 (30 patients, 60 eyes). Age-matched normal subjects with T2D group)	Wide-field contact specular microscope (Tomey EM-1000, Tokyo, Japan)	ECD, mean cell area, CV, CCT, IOP	**T1D** (i) ECD decreased in T1D in comparison with controls. (ii) CCT thicker in T1D in comparison with controls. HbA1c level was inversely correlated with the ECD and correlated with the mean endothelial cell area. (iii) Positive correlation between glucose level and ECD, endothelial cell area and CCT. (iv) Negative correlation between ECD and duration of the disease/insulin therapy. **T2D** (i) No differences were found in the evaluated values compared to controls. No correlations were founded
Urban, 2013 [133]	Case-control Prospective	123 children and adolescents with T1D (123 eyes) 124 controls (124 eyes)	Topcon SP-2000P endothelial microscope.	ECD and CCT	(i) ECD was lower in children-adolescent T1D compared to controls. (ii) CCT was higher in children-adolescent T1D compared to controls. (iii) There was no correlation between ECD and metabolic control, HbA1c level and plasma creatinine level. (iv) Correlation between ECD, CCT, and duration of diabetes was statistically significant.
*Storr-Paulsen, 2014* [134]	*Case-control* *Prospective*	*107 T2D* *128 controls*	*SP 2000P; Topcon, Tokyo, Japan.*	*ECD, CV, hexagonality percentage and CCT*	*(i) No differences between groups.* *(ii) Higher HbA1c was associated with lower ECD.* *(iii) CCT increased in the T2D group.*
*Leelawongtawun, 2015* [135]	*Case-control* *Prospective*	*90 diabetic patients (171 eyes)* *(i) 1 patient (two eyes) with severe NP-DR.* *(ii) 7 patients (11 eyes) with moderate NP-DR* *(iii) 13 patients (24 eyes) with mild NP-DR* *(iv) 71 patients (134 eyes) with no DR* *90 controls (156 eyes).*	*Specular microscope (Confoscan4, Nidek)*	*ECD, percentage of polymegathism, and hexagonality percentage.*	*(i) No differences between diabetes and controls* *(ii) The over one year diabetic patients had a decreased percentage of hexagonal cell compared to controls.* *(iii) The over two years diabetic patients had a decreased percentage of hexagonal cell and an increased percentage of polymegathism compared to controls.*
Calvo-maroto, 2015 [13]	Retrospective	77 noninsulin T2D (77 eyes): (i) Short-term diabetic subjects (recently diagnosed,<1 year since diagnosis) (ii) Long-term diabetic subjects (diagnosed and treated for 10 years or more) s80 controls (80 eyes)	Topcon SP-3000P noncontact specular microscope	CCT, ECD	(i) CCT higher in long-term diabetic patients when compared with short-term diabetic patients and controls. (ii) ECD lower in long-term diabetic patients when compared with short-term diabetic patients and controls.
Szalai, 2016 [12]	Case-control Prospective	28 T1D (28 eyes) 18 with DR 10 without DR 17 age-matched controls (17 eyes)	Corneal confocal microscopy with Heidelberg Retina Tomograph III Rostock Cornea Module (HRT III RCM, Heidelberg Engineering GmbH, Heidelberg, Germany)	ECD Other (epithelial, stromal density. Subbasal nerve morphology)	ECD was lower in T1D with and without DR compared to controls.
Anbar, 2016 [136]	Case-control Prospective	80 T1D children (160 eyes) 40 controls (80 eyes)	Noncontact specular microscope (Topcon SP-1P, Tokyo, Japan).	CCT, ECD, polymegathism, and pleomorphism	(i) CCT higher in the T1D group. (ii) ECD lower in the T1D group. (iii) Percentage of hexagonality lower in the T1D group. Polymegathism higher in the T1D group. (iv) All changes are correlated only with the duration of diabetes
Leelawongtawun, 2016 [137]	Case-control Prospective	148 diabetes (271 eyes). Divided based on diabetes duration (i) Below 5 years (ii) 5 to 10 years (iii) Over 10 years (iv) 46 controls (82 eyes)	Specular microscope (Confoscan4 (CS4), Nidek)	ECD, percentage of polymegathism and hexagonality percentage	(i) ECD was lower in all diabetes groups compared to controls. (ii) In all groups of diabetes, the polymegathism percentage was more than while the hexagonality percentage was less than controls. (iii) There were no differences in all endothelial parameters between 3 groups of diabetes.
Galgauskas, 2016 [77]	Case-control Prospective	62 T2D (123 eyes): (i) 22 (17.9%) eyes with DR (ii) 10 (8.1%) eyes with macular edema 65 controls (120 eyes)	Noncontact specular microscope (SP-9000; Konan Medical Inc., Hyogo, Japan)	CCT, ECD, average size, hexagonality percentage and polymegathism	(i) ECD lower in diabetes than in controls. (ii) CCT higher in diabetes than in controls. (iii) Hemoglobin A1C and the duration of diabetes not associated with any of the examined parameters
El-agamy, 2017 [79]	Case-control Prospective	57 T2D (57 eyes): 36 eyes without DR 14 eyes with NP-DR 7 eyes with P-DR. 45 controls (45 eyes)	EM-3000 Specular Microscope	CCT, ECD, CV and hexagonality percentage	(i) ECD lower in diabetes than in controls. (ii) CV higher in diabetes.
Islam, 2017 [138]	Case-control Prospective	149 diabetes (149 eyes) (i) 52 T1D (ii) I97 T2D 149 controls(149 eyes)	SP–3000P, Topcon Corporation, Japan	ECD, average cell size, CV and hexagonality percentage	(i) ECD lower in the diabetes group Diabetes longer than 10 years had significantly lower ECD and larger average size. (ii) Diabetes duration was correlated with ECD, polymegethism and hexagonality
*Qu, 2017* [13]	*Case-control* *Prospective*	*87 T2D (87 eyes): (i) 48 eyes without cornea fluorescein staining* *(ii) 39 eyes with cornea fluorescein staining* *51 controls (51 eyes)*	*Keratograph 5M (K5M; OCULUS Optikgerate GmbH, Wetzlar, Germany)*	*Basal epithelial cell density, subbasal nerve plexus density, langerhans cell density and ECD.*	*No differences in ECD between groups*

T1D: type 1 diabetes; T2D: type 2 diabetes; DR: diabetic retinopathy; NP-DR: nonproliferative diabetic retinopathy; P-DR: proliferative diabetic retinopathy; ECD: endothelial cell density; CV: coefficient of variation of cell area; CCT: central corneal thickness; IOP: intraocular pressure.

**Table 3:** Summary of “in vitro” studies of the effect of diabetes on the corneal endothelium.

Author, year	Type of study	Study groups	Technology	Parameters	Results
Kwon, 2016 [143]	Descriptive	18,665 donors (34,234 corneas)	Specular microscopy (Konan Cell Chek EB-10; Konan Medical, Hyogo, Japan)	(i) Sex, age, race, surgery, disease (hypertension, diabetes, glaucoma, depression, dementia, Parkinson, hyperthyroidism and hypothyroidism) and habits (smokers/nonsmokers) (ii) All independent variables were divided into 2 groups: (1) ECD>2000 cels/mm^2^ (2) ECD<2000 cels/mm^2^	(i) ECD decreased with age. (ii) The average ECD of African American donors was higher than those of white or Hispanic donors. (iii) A history of diabetes and ocular surgery were associated with a lower ECD. (iv) Age, history of cataract surgery and diabetes were found to be the greatest risk factors for inadequate donor quality with respect to ECD.
Schwarz, 2016 [144]	Case-control	22 donors (27 corneas): (i) Nondiabetes (9 corneas, 8 donors) (ii) Diabetes without evidence of advanced disease (8 corneas, 7 donors) (iii) Diabetes with evidence of advanced disease (10 corneas, 7 donors).	(i) Specular microscopy (technology not specified) (ii) The adhesion strength of endothelium-descemet membrane complex to the posterior stroma was measured by an own method developed by the investigators (see article).	(i) ECD, hexagonality, and CV. (ii) Variables obtained from mechanical peel testing were: (1) Endothelium-descemet membrane complex elastic peel tension (TE) (2) Elastic stiffness (SE) (3) Average delamination tension (TD), and maximum tension (TMAX)	(i) The three groups did not differ in ECD, hexagonality, and CV. (ii) Diabetes with evidence of advanced disease had values for TE, TD, and TMAX greater than nondiabetes and diabetes without evidence of advanced disease corneas.
Liaboe, 2017 [145]	Retrospective case-controls	2112 donors (4185 corneas) divided in 4 groups: (i) Nondiabetes(2636 corneas) (ii) NID-diabetes (847 corneas) (iii) ID-diabetes without medical complications due to diabetes (471 corneas) (iv) I-diabetes with medical complications due to diabetes (231 corneas).	Noncontact specular microscopy (KeratoAnalyzer EKA-10; Konan Medical USA, Irvine, CA)	Donor age, death to preservation time, ECD, hexagonality, and CV.	(i) I-diabetes with medical complications due to diabetes corneas showed a significant reduction in mean ECD compared with nondiabetic and NI-diabetes. (ii) There were no significant differences in endothelial cell hexagonality or coefficient of variation among the 4 groups.
Aldrich, 2017 [146]	Case-control	159 donors (229 corneas) all of them with ECD> 2000 cells/mm^2^. Divided in 4 groups: (i) Nondiabetes (ii) NID-diabetes (iii) ID-diabetes without medical complications due to diabetes (iv) ID-diabetes with medical complications due to diabetes	(i) Noncontact specular microscopy (KeratoAnalyzer EKA-10; Konan Medical USA, Irvine, CA, USA) (ii) Transmission electron microscopes (EM 906E; Carl Zeiss Microscopy, Oberkochen, Germany)	(i) ECD, hexagonality, and CV. (ii) Qualitative and quantitative ultrastructural changes in corneal endothelial cells quantified with transmission electron microscope: (iii) Number of mitochondria per µm^2^, surface area per mitochondria in µm^2^, and total mitochondrial surface area per 20 µm^2^ field of view.	(i) ID-diabetes with medical complications due to diabetes displayed the lowest spare respiratory values compared to all other groups. (ii) The remaining mitochondrial respiration and glycolysis metrics did not differ significantly among groups. (iii) Compared to nondiabetes, the endothelium from ID-diabetes with medical complications due to diabetes had alterations in mitochondrial morphology, pronounced Golgi bodies associated with abundant vesicles, accumulation of lysosomal bodies/autophagosomes, and focal production of abnormal long-spacing collagen.
Chen, 2017 [147]	Case-control	(i) 20,026 nondiabetes donor eyes (ii) 13,617 diabetes donor eyes	Specular microscope (Konan EB-10; Konan, Hyogo, Japan).	ECD	Amongst phakic donors, diabetic ECD was lower in the middle aged subgroups, between 21 and 40 years and between 41 and 60 years. There was no difference in ECD for phakic corneas from the subset aged 61 years or older.
Chocron, 2018 [148]	Retrospective case-control	17056 donors: (i) Diabetes (4766 patients): (ii) Metformin consumers (iii) Nonmetformin consumers (iv) Controls (12290 patients)	Specular microscopy (Konan Cell Check EB-10; Konan, Hyogo, Japan)	Age, sex, race, medical history, medication list at the time of death, and ECD.	(i) ECD was lower in patients with diabetes. (ii) ECD was not associated with metformin use in patients with diabetes. (iii) Metformin use was significantly associated with lower ECD among patients with glaucoma.
Skeie, 2018 [149]	Case-control	19 donors: (i) 4 nondiabetes (ii) 10 nonadvanced diabetes (without or with history of home insulin use) (iii) 5 advanced diabetes with medical complications due to diabetes (history of home insulin use and end-organ damage specifically noted in the medical history)	Multidimensional protein identification technology mass spectrometry	Corneal endothelial cell layer and descemet membrane proteome characterization	(i) Decrease in relative protein abundance in insulin-dependent samples (nonadvanced diabetes insulin-dependent and advanced diabetes) compared to non-insulin-dependent samples (nondiabetes and nonadvanced diabetes without insulin use). (ii) Comparing the nonadvanced diabetes insulin-dependent and advanced diabetes groups, mitochondria protein levels appear to increase as the disease progresses.

NID-diabetes: non-insulin-dependent diabetes mellitus; ID-diabetes: insulin-dependent diabetes mellitus. ECD: endothelial cell density; CV: coefficient of variation of cell area.

## References

[1] World Health Organization (2016). *Global Report on Diabetes*.

[2] Han S. B., Yang H. K., Hyon J. Y. (2018). Influence of diabetes mellitus on anterior segment of the eye. *Clinical Interventions in Aging*.

[3] Bikbova G., Oshitari T., Baba T., Bikbov M., Yamamoto S. (2018). Diabetic corneal neuropathy: clinical perspectives. *Clinical Ophthalmology*.

[4] Ljubimov A. V. (2017). Diabetic complications in the cornea. *Vision Research*.

[5] Bikbova G., Oshitari T., Baba T., Yamamoto S. (2016). Neuronal changes in the diabetic cornea: perspectives for neuroprotection. *BioMed Research International*.

[6] Misra S. L., Braatvedt G. D., Patel D. V. (2016). Impact of diabetes mellitus on the ocular surface: a review. *Clinical & Experimental Ophthalmology*.

[7] Lv H., Li A., Zhang X. (2014). Meta-analysis and review on the changes of tear function and corneal sensitivity in diabetic patients. *Acta Ophthalmologica*.

[8] Liu H., Sheng M., Liu Y (2015). Expression of SIRT1 and oxidative stress in diabetic dry eye. *International Journal of Clinical and Experimental Pathology*.

[9] Shih K. C., Lam K. S.-L., Tong L. (2017). A systematic review on the impact of diabetes mellitus on the ocular surface. *Nutrition & Diabetes*.

[10] Cai D., Zhu M., Petroll W. M., Koppaka V., Robertson D. M. (2014). The impact of type 1 diabetes mellitus on corneal epithelial nerve morphology and the corneal epithelium. *American Journal of Pathology*.

[11] Dehghani C., Pritchard N., Edwards K., Russell A. W., Malik R. A., Efron N. (2016). Abnormal anterior corneal morphology in diabetes observed using in vivo laser-scanning confocal microscopy. *The Ocular Surface*.

[12] Szalai E., Deák E., Módis L. (2016). Early corneal cellular and nerve fiber pathology in young patients with type 1 diabetes mellitus identified using corneal confocal microscopy. *Investigative Opthalmology & Visual Science*.

[13] Qu J. H., Li L., Tian L., Zhang X. Y., Thomas R., Sun X. G. (2018). Epithelial changes with corneal punctate epitheliopathy in type 2 diabetes mellitus and their correlation with time to healing. *BMC Ophthalmology*.

[14] Quadrado M. J., Popper M., Morgado A. M., Murta J. N., Van Best J. A. (2006). Diabetes and corneal cell densities in humans by in vivo confocal microscopy. *Cornea*.

[15] Taylor H. R., Kimsey R. A. (1981). Corneal epithelial basement membrane changes in diabetes. *Investigative Ophthalmology & Visual Science*.

[16] Sato N., Nakamura M., Chikama T., Nishida T. (1999). Abnormal deposition of laminin and type IV collagen at corneal epithelial basement membrane during wound healing in diabetic rats. *Japanese Journal of Ophthalmology*.

[17] Ljubimov A. V., Huang Z.-S., Huang G. H. (1998). Human corneal epithelial basement membrane and integrin alterations in diabetes and diabetic retinopathy1. *Journal of Histochemistry & Cytochemistry*.

[18] Foulks G. N., Thoft R. A., Perry H. D., Tolentino F. I. (1979). Factors related to corneal epithelial complications after closed vitrectomy in diabetics. *Archives of Ophthalmology*.

[19] Morishige N., Chikama T.-I., Sassa Y., Nishida T. (2001). Abnormal light scattering detected by confocal biomicroscopy at the corneal epithelial basement membrane of subjects with type II diabetes. *Diabetologia*.

[20] Takah Takahashi N., Wakuta M., Morishige N., Chikama T., Nishida T., Sumii Y. (2007). Development of an instrument for measurement of light scattering at the corneal epithelial basement membrane in diabetic patients. *Japanese Journal of Ophthalmology*.

[21] Özyol P., Özyol E. (2018). Assessment of corneal backward light scattering in diabetic patients. *Eye & Contact Lens: Science & Clinical Practice*.

[22] Ljubimov A. V., Burgeson R. E., Butkowski R. J. (1996). Basement membrane abnormalities in human eyes with diabetic retinopathy. *Journal of Histochemistry & Cytochemistry*.

[23] Saghizadeh M., Kramerov A. A., Yaghoobzadeh Y. (2010). Adenovirus-driven overexpression of proteinases in organ-cultured normal human corneas leads to diabetic-like changes. *Brain Research Bulletin*.

[24] Saghizadeh M., Soleymani S., Harounian A. (2011). Alterations of epithelial stem cell marker patterns in human diabetic corneas and effects of c-met gene therapy. *Molecular Vision*.

[25] Grushkin-Lerner L. S., Kewalramani R., Trinkaus-Randall V. (1997). Expression of integrin receptors on plasma membranes of primary corneal epithelial cells is matrix specific. *Experimental Eye Research*.

[26] Huang C., Liao R., Wang F., Tang S. (2016). Characteristics of reconstituted tight junctions after corneal epithelial wounds and ultrastructure alterations of corneas in type 2 diabetic rats. *Current Eye Research*.

[27] Yin J., Huang J., Chen C., Gao N., Wang F., Yu F.-S. X. (2011). Corneal complications in streptozocin-induced type I diabetic rats. *Investigative Opthalmology & Visual Science*.

[28] Shi L., Chen H., Yu X., Wu X. (2013). Advanced glycation end products delay corneal epithelial wound healing through reactive oxygen species generation. *Molecular and Cellular Biochemistry*.

[29] Shi L., Yu X., Yang H., Wu X. (2013). Advanced glycation end products induce human corneal epithelial cells apoptosis through generation of reactive oxygen species and activation of JNK and p38 MAPK pathways. *PLoS One*.

[30] Kim J., Kim C.-S., Sohn E., Jeong I.-H., Kim H., Kim J. S. (2011). Involvement of advanced glycation end products, oxidative stress and nuclear factor-kappaB in the development of diabetic keratopathy. *Graefe’s Archive for Clinical and Experimental Ophthalmology*.

[31] Kim J., Kim C.-S., Kim H., Jeong I.-H., Sohn E., Kim J. S. (2011). Protection against advanced glycation end products and oxidative stress during the development of diabetic keratopathy by KIOM-79. *Journal of Pharmacy and Pharmacology*.

[32] Zou C., Wang S., Huang F., Zhang Y. A. (2012). Advanced glycation end products and ultrastructural changes in corneas of long-term streptozotocin-induced diabetic monkeys. *Cornea*.

[33] Zhao Z., Liu J., Shi B., He S., Yao X., Willcox M. D (2010). Advanced glycation end product (AGE) modified proteins in tears of diabetic patients. *Molecular Vision*.

[34] Xu K., Yu F.-S. X. (2011). Impaired epithelial wound healing and EGFR signaling pathways in the corneas of diabetic rats. *Investigative Opthalmology & Visual Science*.

[35] Sun H., Mi X., Gao N., Yan C., Yu F.-S. (2015). Hyperglycemia-suppressed expression of serpine1 contributes to delayed epithelial wound healing in diabetic mouse corneas. *Investigative Opthalmology & Visual Science*.

[36] Ljubimov A. V., Saghizadeh M. (2015). Progress in corneal wound healing. *Progress in Retinal and Eye Research*.

[37] Yin J., Yu F.-S. X. (2010). LL-37 via EGFR transactivation to promote high glucose-attenuated epithelial wound healing in organ-cultured corneas. *Investigative Opthalmology & Visual Science*.

[38] Xu K.-P., Li Y., Ljubimov A. V., Yu F.-S. X. (2009). High glucose suppresses epidermal growth factor receptor/phosphatidylinositol 3-kinase/akt signaling pathway and attenuates corneal epithelial wound healing. *Diabetes*.

[39] Kakazu A., Sharma G., Bazan H. E. P. (2008). Association of protein tyrosine phosphatases (PTPs)-1B with c-Met receptor and modulation of corneal epithelial wound healing. *Investigative Opthalmology & Visual Science*.

[40] Saika S., Okada Y., Miyamoto T. (2004). Role of p38 MAP kinase in regulation of cell migration and proliferation in healing corneal epithelium. *Investigative Opthalmology & Visual Science*.

[41] Saghizadeh M., Kramerov A. A., Yu F.-S. X., Castro M. G., Ljubimov A. V. (2010). Normalization of wound healing and diabetic markers in organ cultured human diabetic corneas by adenoviral delivery ofc-metgene. *Investigative Opthalmology & Visual Science*.

[42] Saghizadeh M., Dib C. M., Brunken W. J., Ljubimov A. V. (2014). Normalization of wound healing and stem cell marker patterns in organ-cultured human diabetic corneas by gene therapy of limbal cells. *Experimental Eye Research*.

[43] Saghizadeh M., Epifantseva I., Hemmati D. M., Ghiam C. A., Brunken W. J., Ljubimov A. V. (2013). Enhanced wound healing, kinase and stem cell marker expression in diabetic organ-cultured human corneas upon MMP-10 and cathepsin F gene silencing. *Investigative Opthalmology & Visual Science*.

[44] Kramerov A. A., Saghizadeh M., Ljubimov A. V. (2016). Adenoviral gene therapy for diabetic keratopathy: effects on wound healing and stem cell marker expression in human organ-cultured corneas and limbal epithelial cells. *Journal of Visualized Experiments*.

[45] McLaughlin P. J., Sassani J. W., Klocek M. S., Zagon I. S. (2010 Feb 15). Diabetic keratopathy and treatment by modulation of the opioid growth factor (OGF)-OGF receptor (OGFr) axis with naltrexone: a review. *Brain Research Bulletin*.

[46] Sassani J. W., Mc Laughlin P. J., Zagon I. S. (2016). The Yin and Yang of the opioid growth regulatory system: focus on diabetes-the Lorenz E. Zimmerman tribute lecture. *Journal of Diabetes Research*.

[47] Wu Y.-C., Buckner B. R., Zhu M., Cavanagh H. D., Robertson D. M. (2012). Elevated IGFBP3 levels in diabetic tears: a negative regulator of IGF-1 signaling in the corneal epithelium. *The Ocular Surface*.

[48] Wang Y., Zhao X., Shi D. (2013). Overexpression of SIRT1 promotes high glucose-attenuated corneal epithelial wound healing via p53 regulation of the IGFBP3/IGF-1R/AKT pathway. *Investigative Opthalmology & Visual Science*.

[49] Gao J., Wang Y., Zhao X., Chen P., Xie L. (2015). MicroRNA-204-5p-Mediated regulation of SIRT1 contributes to the delay of epithelial cell cycle traversal in diabetic corneas. *Investigative Ophthalmology & Visual Science*.

[50] Shen Y., Pfluger T., Ferreira F. (2016). Diabetic cornea wounds produce significantly weaker electric signals that may contribute to impaired healing. *Scientific Reports*.

[51] Müller L. J., Marfurt C. F., Kruse F., Tervo T. M. T. (2003). Corneal nerves: structure, contents and function. *Experimental Eye Research*.

[52] He J., Bazan N. G., Bazan H. E. P. (2010). Mapping the entire human corneal nerve architecture. *Experimental Eye Research*.

[53] Marfurt C. F., Cox J., Deek S., Dvorscak L. (2010). Anatomy of the human corneal innervation. *Experimental Eye Research*.

[54] Inoue K., Okugawa K., Amano S. (2005). Blinking and superficial punctate keratopathy in patients with diabetes mellitus. *Eye*.

[55] Garcia-Hirschfeld J., Lopez-Briones L. G., Belmonte C. (1994). Neurotrophic influences on corneal epithelial cells. *Experimental Eye Research*.

[56] Millodot M. (1984). A review of research on the sensitivity of the cornea. *Ophthalmic and Physiological Optics*.

[57] Dartt D. A. (2004). Dysfunctional neural regulation of lacrimal gland secretion and its role in the pathogenesis of dry eye syndromes. *The Ocular Surface*.

[58] Nishida T., Chikama T.-I., Sawa M., Miyata K., Matsui T., Shigeta K. (2012). Differential contributions of impaired corneal sensitivity and reduced tear secretion to corneal epithelial disorders. *Japanese Journal of Ophthalmology*.

[59] Zhivov A., Winter K., Hovakimyan M. (2013). Imaging and quantification of subbasal nerve plexus in healthy volunteers and diabetic patients with or without retinopathy. *PLoS One*.

[60] Pritchard N., Edwards K., Russell A. W., Perkins B. A, Malik R. A, Efron N (2015). Corneal confocal microscopy predicts 4-year incident peripheral neuropathy in type 1 diabetes. *Diabetes Care*.

[61] Mocan M. C., Durukan I., Irkec M., Orhan M. (2006). Morphologic alterations of both the stromal and subbasal nerves in the corneas of patients with diabetes. *Cornea*.

[62] Cillà S. D., Ranno S., Carini E. (2009). Corneal subbasal nerves changes in patients with diabetic retinopathy: an in vivo confocal study. *Investigative Opthalmology & Visual Science*.

[63] Yang A. Y., Chow J., Liu J. (2018). Corneal innervation and sensation: the eye and beyond. *Yale Journal of Biology and Medicine*.

[64] He J., Bazan H. E. P. (2012). Mapping the nerve architecture of diabetic human corneas. *Ophthalmology*.

[65] Abdelkader H., Patel D. V., McGhee C. N., Alany R. G. (2011). New therapeutic approaches in the treatment of diabetic keratopathy: a review. *Clinical & Experimental Ophthalmology*.

[66] O’Donnell C., Efron N. (2012). Diabetes and contact lens wear. *Clinical & Experimental Optometry*.

[67] Pritchard N., Edwards K., Shahidi A. M. (2011). Corneal markers of diabetic neuropathy. *The Ocular Surface*.

[68] Alam U., Jeziorska M., Petropoulos I. N. (2017). Diagnostic utility of corneal confocal microscopy and intra-epidermal nerve fibre density in diabetic neuropathy. *PLoS One*.

[69] Chen X., Graham J., Dabbah M. A. (2015). Small nerve fiber quantification in the diagnosis of diabetic sensorimotor polyneuropathy: comparing corneal confocal microscopy with intraepidermal nerve fiber density. *Diabetes Care*.

[70] Asghar O., Petropoulos I. N., Alam U. (2014). Corneal confocal microscopy detects neuropathy in subjects with impaired glucose tolerance: figure 1. *Diabetes Care*.

[71] Tavakoli M., Mitu-Pretorian M., Petropoulos I. N. (2013). Corneal confocal microscopy detects early nerve regeneration in diabetic neuropathy after simultaneous pancreas and kidney transplantation. *Diabetes*.

[72] Qu J. H., Tian L., Zhang X. Y., Sun X. G. (2017). Early central and peripheral corneal microstructural changes in type 2 diabetes mellitus patients identified using in vivo confocal microscopy: a case-control study. *Medicine (Baltimore)*.

[73] Česká Burdová M., Kulich M., Dotřelová D., Mahelková G. (2018). Effect of diabetes mellitus type 1 diagnosis on the corneal cell densities and nerve fibers. *Physiological Research*.

[74] Hager A., Wegscheider K., Wiegand W. (2009). Changes of extracellular matrix of the cornea in diabetes mellitus. *Graefe’s Archive for Clinical and Experimental Ophthalmology*.

[75] Robert L., Legeais J. M., Robert A. M., Renard G. (2001). Corneal collagens. *Pathologie Biologie*.

[76] Kotecha A. (2007). What biomechanical properties of the cornea are relevant for the clinician?. *Survey of Ophthalmology*.

[77] Galgauskas S., Laurinavičiūtė G., Norvydaitė D., Stech S., Ašoklis R. (2016). Changes in choroidal thickness and corneal parameters in diabetic eyes. *European Journal of Ophthalmology*.

[78] Briggs S., Osuagwu U. L., AlHarthi E. M. (2015). Manifestations of type 2 diabetes in corneal endothelial cell density, corneal thickness and intraocular pressure. *Journal of Biomedical Research*.

[79] El-Agamy A., Alsubaie S. (2017). Corneal endothelium and central corneal thickness changes in type 2 diabetes mellitus. *Clinical Ophthalmology*.

[80] Sanchis-Gimeno J. A., Alonso L., Rahhal M., Bastir M., Perez-Bermejo M., Belda-Salmeron L. (2017). Corneal thickness differences between type 2 diabetes and non-diabetes subjects during preoperative laser surgery examination. *Journal of Diabetes and its Complications*.

[81] Toygar O., Sizmaz S., Peli̇t A., Toygar B., Yabaş Kiziloğlu Ö., Akova Y. (2015). Central corneal thickness in type II diabetes mellitus: is it related to the severity of diabetic retinopathy?. *Turkish Journal of Medical Sciences*.

[82] Ozdamar Y., Cankaya B., Ozalp S., Acaroglu G., Karakaya J., Özkan S. S. (2010). Is there a correlation between diabetes mellitus and central corneal thickness?. *Journal of Glaucoma*.

[83] Ni S., Yu J., Bao F., Li J., Elsheikh A., Wang Q. (2011). Effect of glucose on the stress-strain behavior of ex-vivo rabbit cornea. *Experimental Eye Research*.

[84] Nishitsuka K., Kawasaki R., Kanno M. (2011). Determinants and risk factors for central corneal thickness in Japanese persons: the Funagata study. *Ophthalmic Epidemiology*.

[85] Suraida A. R., Ibrahim M., Zunaina E. (2018). Correlation of the anterior ocular segment biometry with HbA1c level in type 2 diabetes mellitus patients. *PLoS One*.

[86] Santiagu F., Bakhtiari A., Iqbal T., Khaliddin N., Lansingh V. C., Subrayan V. (2018). Diabetes and pachymetry changes in pregnancy. *International Ophthalmology*.

[87] Kumar N., Pop-Busui R., Musch D. C. (2018). Central corneal thickness increase due to stromal thickening with diabetic peripheral neuropathy severity. *Cornea*.

[88] Adnan X., Suheimat M., Efron N. (2015). Biometry of eyes in type 1 diabetes. *Biomedical Optics Express*.

[89] Soleimanizad R., Nowroozzadeh M. H., Ziaei H. (2017). The association of central corneal thickness with ocular and general parameters in a community setting: the yazd eye study. *Journal of Ophthalmic & Vision Research*.

[90] Sng C., Barton K., Kim H., Yuan S., Budenz D. L. (2016). Central corneal thickness and its associations with ocular and systemic factors in an urban west african population. *American Journal of Ophthalmology*.

[91] Schweitzer C., Korobelnik J.-F., Boniol M. (2016). Associations of biomechanical properties of the cornea with Environmental and metabolic factors in an elderly population: the ALIENOR Study. *Investigative Opthalmology & Visual Science*.

[92] Castro D. P. E., Prata T. S., Lima V. C., Biteli L. G., de Moraes C. G., Paranhos A. (2010). Corneal viscoelasticity differences between diabetic and nondiabetic glaucomatous patients. *Journal of Glaucoma*.

[93] Akkaya S., Can E., Ö naya F. (2016). Comparison of the corneal biomechanical properties, optic nerve head topographic parameters, and retinal nerve fiber layer thickness measurements in diabetic and non-diabetic primary open-angle glaucoma. *International Ophthalmology*.

[94] Hashemi H., Asgari S., Mehravaran S., Emamian M. H., Fotouhi A. (2018). Five-year changes of anterior corneal indices in diabetics versus non-diabetics: the shahroud eye cohort study. *Current Eye Research*.

[95] Tiutiuca C. (2013). Assessment of central corneal thickness in children with diabetes mellitus type I. *Oftalmologia*.

[96] Akinci A., Bulus D., Aycan Z., Oner O. (2009). Central corneal thickness in children with diabetes. *Journal of Refractive Surgery*.

[97] Uzel M. M., Elgin U., Sen E., Keskin M., Sağsak E., Aycan Z. (2016). Comparison of anterior segment parameters in juvenile diabetes mellitus and healthy eyes. *European Journal of Ophthalmology*.

[98] Akil H., Buluş A., Andiran N., Alp M. (2016). Ocular manifestations of type 1 diabetes mellitus in pediatric population. *Indian Journal of Ophthalmology*.

[99] Kara N., Yildirim Y., Univar T., Kontbay T. (2013). Corneal biomechanical properties in children with diabetes mellitus. *European Journal of Ophthalmology*.

[100] Jeziorny K., Niwald A., Moll A. (2018). Measurement of corneal thickness, optic nerve sheath diameter and retinal nerve fiber layer as potential new non-invasive methods in assessing a risk of cerebral edema in type 1 diabetes in children. *Acta Diabetologica*.

[101] Luce D. A. (2005). Determining in vivo biomechanical properties of the cornea with an ocular response analyzer. *Journal of Cataract & Refractive*.

[102] Dupps W. J., Wilson S. E. (2006). Biomechanics and wound healing in the cornea. *Experimental Eye Research*.

[103] Roberts C. (2000). The cornea is not a piece of plastic. *Journal of Refractive Surgery*.

[104] Shah S., Laiquzzaman M., Bhojwani R., Mantry S., Cunliffe I. (2007). Assessment of the biomechanical properties of the cornea with the ocular response analyzer in normal and keratoconic eyes. *Investigative Opthalmology & Visual Science*.

[105] del Buey M. A. A., Cristóbal J. A., Ascaso F. J., Lavilla L., Lanchares E. (2009). Biomechanical properties of the cornea in Fuchs’ corneal dystrophy. *Investigative Opthalmology & Visual Science*.

[106] Brown K. E., Congdon N. G. (2006). Corneal structure and biomechanics: impact on the diagnosis and management of glaucoma. *Current Opinion in Ophthalmology*.

[107] Chihara E. (2008). Assessment of true intraocular pressure: the gap between theory and practical data. *Survey of Ophthalmology*.

[108] Goldich Y., Barkana Y., Gerber Y. (2009). Effect of diabetes mellitus on biomechanical parameters of the cornea. *Journal of Cataract & Refractive Surgery*.

[109] Sahin A., Bayer A., Ozge G., Mumcuoglu T. (2009). Corneal biomechanical changes in diabetes mellitus and their influence on intraocular pressure measurements. *Investigative Ophthalmology & Visual Science*.

[110] Kotecha A., Oddone F., Sinapis C. (2010). Corneal biomechanical characteristics in patients with diabetes mellitus. *Journal of Cataract & Refractive Surgery*.

[111] Nalcacioglu-Yuksekkaya P., Sen E., Cetinkaya S., Bas V., Aycan Z., Ozturk F. (2014). Corneal biomechanical characteristics in children with diabetes mellitus. *International Ophthalmology*.

[112] Celik U., Yazgan S., Orhan A. (2014). Evaluation of the relationship between corneal biomechanic and HbA1C levels in type 2 diabetes patients. *Clinical Ophthalmology*.

[113] Perez-Rico C., Gutierrez-Ortiz C., Gonzalez-Mesa A. (2015). Effect of diabetes mellitus on corvis ST measurement process. *Acta Ophthalmologica*.

[114] Bekmez S., Kocaturk T. (2018). Higher intraocular pressure levels associated with lower hysteresis in type 2 diabetes. *Open Ophthalmology Journal*.

[115] Scheler A., Spoerl E., Boehm A. G. (2012). Effect of diabetes mellitus on corneal biomechanics and measurement of intraocular pressure. *Acta Ophthalmologica*.

[116] Kosker M., Suri K., Hammersmith K. M., Nassef A. H., Nagra P. K., Rapuano C. J. (2014). Another look at the association between diabetes and keratoconus. *Cornea*.

[117] Narayanaswamy A., Chung R. S., Wu R. Y. (2011). Determinants of corneal biomechanical properties in an adult Chinese population. *Ophthalmology*.

[118] Naderan M., Naderan M., Rezagholizadeh F., Zolfaghari M., Pahlevani R., Rajabi M. T. (2014). Association between diabetes and keratoconus. *Cornea*.

[119] Hussnain S. A., Alsberge J. B., Ehrlich J. R., Shimmyo M., Radcliffe N. M. (2015). Change in corneal hysteresis over time in normal, glaucomatous and diabetic eyes. *Acta Ophthalmol*.

[120] Luo X.-Y., Tan N. Y. Q., Chee M.-L. (2018). Direct and indirect associations between diabetes and intraocular pressure: the Singapore epidemiology of eye diseases study. *Investigative Opthalmology & Visual Science*.

[121] Cennamo G., Forte R., Aufiero B., La Rana A. (2011). Computerized Scheimpflug densitometry as a measure of corneal optical density after excimer laser refractive surgery in myopic eyes. *Journal of Cataract & Refractive Surgery*.

[122] Takacs A. I., Mihaltz K., Nagy Z. Z. (2011). Corneal density with the pentacam after photorefractive keratectomy. *Journal of Refractive Surgery*.

[123] Gao F., Lin T., Pan Y. (2016). Effects of diabetic keratopathy on corneal optical density, central corneal thickness, and corneal endothelial cell counts. *Experimental and Therapeutic Medicine*.

[124] Calvo-Maroto A. M., Pérez-Cambrodí R. J., Esteve-Taboada J. J., García-Lázaro S., Cerviño A. N. (2017). Corneal backscatter in insulin-dependent and non-insulin-dependent diabetes mellitus patients: a pilot study. *Arquivos Brasileiros de Oftalmologia*.

[125] Tekin K., Inanc M., Kurnaz E. (2017). Objective evaluation of corneal and lens clarity in children with type 1 diabetes mellitus. *American Journal of Ophthalmology*.

[126] Bao F., Deng M., Zheng X. (2017). Effects of diabetes mellitus on biomechanical properties of the rabbit cornea. *Experimental Eye Research*.

[127] Goldin A., Beckman J. A., Schmidt A. M., Creager M. A. (2006). Advanced glycation end products. *Circulation*.

[128] del Buey M. A., Lavilla L., AscasoFJ (2014). Assessment of corneal biomechanical properties and intraocular pressure in myopic Spanish healthy population. *Journal of Ophthalmology*.

[129] Luz A., Lopes B., Hallahan K. M. (2016). Enhanced combined tomography and biomechanics data for distinguishing forme fruste keratoconus. *Journal of Refractive Surgery*.

[130] Long Q., Wang J. Y., Xu D. (2017). Comparison of corneal biomechanics in sjögren’s syndrome and non-Sjögren’s syndrome dry eyes by Scheimpflug based device. *International Journal of Ophthalmology*.

[131] Shenoy R., Khandekar R., Bialasiewicz A. A., Muniri A. A. (2009). Corneal endothelium in patients with diabetes mellitus: a historical cohort study. *European Journal of Ophthalmology*.

[132] Módis L., Szalai E., Kertész K., Kemény-Beke A., Kettesy B., Berta A. (2010). Evaluation of the corneal endothelium in patients with diabetes mellitus type I and II. *Histology and Histopathology*.

[133] Urban B., Raczyńska D., Bakunowicz-Łazarczyk A., Raczyńska K., Krętowska M. (2013). Evaluation of corneal endothelium in children and adolescents with type 1 diabetes mellitus. *Mediators of Inflammation*.

[134] Storr-Paulsen A., Singh A., Jeppesen H., Norregaard J. C., Thulesen J. (2014). Corneal endothelial morphology and central thickness in patients with type II diabetes mellitus. *Acta Ophthalmologica*.

[135] Leelawongtawun W., Suphachearaphan W., Kampitak K., Leelawongtawun R. (2015). A comparative study of corneal endothelial structure between diabetes and non-diabetes. *Journal of the Medical Association of Thailand*.

[136] Anbar M., Ammar H., Mahmoud R. A. (2016). Corneal endothelial morphology in children with type 1 diabetes. *Journal of Diabetes Research*.

[137] Leelawongtawun W., Surakiatchanukul B., Kampitak K., Leelawongtawun R. (2016). Study of corneal endothelial cells related to duration in diabetes. *Journal of the Medical Association of Thailand*.

[138] Islam Q. U., Mehboob M. A., Amin Z. A. (2017). Comparison of corneal morphological characteristics between diabetic and non diabetic population. *Pakistan Journal of Medical Sciences*.

[139] Calvo-Maroto A. M., Cerviño A., Perez-Cambrodí R. J., García-Lázaro S., Sanchis-Gimeno J. A. (2015). Quantitative corneal anatomy: evaluation of the effect of diabetes duration on the endothelial cell density and corneal thickness. *Ophthalmic and Physiological Optics*.

[140] Güell J. L., El Husseiny M. A., Manero F., Gris O., Elies D. (2014). Historical review and update of surgical treatment for corneal endothelial diseases. *Ophthalmology and Therapy*.

[141] Ho J. W., Afshari N. A. (2015). Advances in cataract surgery. *Current Opinion in Ophthalmology*.

[142] Rosado-Adames N., Afshari N. A. (2012). The changing fate of the corneal endothelium in cataract surgery. *Current Opinion in Ophthalmology*.

[143] Kwon J. W., Cho K. J., Kim H. K. (2016 Sep). Analyses of factors affecting endothelial cell density in an eye bank corneal donor database. *Cornea*.

[144] Schwarz C., Aldrich B. T., Burckart K. A. (2016 Dec). Descemet membrane adhesion strength is greater in diabetics with advanced disease compared to healthy donor corneas. *Experimental Eye Research*.

[145] Liaboe C. A., Aldrich B. T., Carter P. C. (2017). Assessing the impact of diabetes mellitus on donor corneal endothelial cell density. *Cornea*.

[146] Aldrich B. T., Schlötzer-Schrehardt U., Skeie J. M. (2017 Apr 1). Mitochondrial and morphologic alterations in native human corneal endothelial cells associated with diabetes mellitus. *Investigative Opthalmology & Visual Science*.

[147] Chen Y., Tsao S. W., Heo M (2017). Age-stratified analysis of diabetes and pseudophakia effects on corneal endothelial cell density: a retrospective eye bank study. *Cornea*.

[148] Chocron I. M., Rai D. K., Kwon J.-W. (2018). Effect of diabetes mellitus and metformin on central corneal endothelial cell density in eye bank eyes. *Cornea*.

[149] Skeie J. M., Aldrich B. T., Goldstein A. S. (2018). Proteomic analysis of corneal endothelial cell-descemet membrane tissues reveals influence of insulin dependence and disease severity in type 2 diabetes mellitus. *PLoS One*.

[150] Han S. B., Yang H. K., Hyon J. Y., Wee W. R. (2017). Association of dry eye disease with psychiatric or neurological disorders in elderly patients. *Clinical Interventions in Aging*.

[151] Goyal S., Hamrah P. (2016). Understanding neuropathic corneal pain-gaps and current therapeutic approaches. *Seminars in Ophthalmology*.

[152] Wang C., Peng Y., Pan S., Li L. (2014). Effect of insulin-like growth factor-1 on corneal surface ultrastructure and nerve regeneration of rabbit eyes after laser in situ keratomileusis. *Neuroscience Letters*.

[153] Chikamoto N., Chikama T.-i., Yamada N., Nishida T., Ishimitsu T., Kamiya A. (2009). Efficacy of substance P and insulin-like growth factor-1 peptides for preventing postsurgical superficial punctate keratopathy in diabetic patients. *Japanese Journal of Ophthalmology*.

[154] Markoulli M., Flanagan J., Tummanapalli S. S., Wu J., Willcox M. (2018). The impact of diabetes on corneal nerve morphology and ocular surface integrity. *The Ocular Surface*.

[155] Sosne G., Rimmer D., Kleinman H. K., Ousler G. (2016). Thymosin beta 4. *Vitamins and Hormones*.

[156] Park J. H., Kang S.-S., Kim J. Y., Tchah H. (2016). Nerve growth factor attenuates apoptosis and inflammation in the diabetic cornea. *Investigative Opthalmology & Visual Science*.

[157] Shevalye H., Yorek M. S., Coppey L. J. (2015). Effect of enriching the diet with menhaden oil or daily treatment with resolvin D1 on neuropathy in a mouse model of type 2 diabetes. *Journal of Neurophysiology*.

[158] Kim S.-Y., Choi J.-S., Joo C.-K. (2009). Effects of nicergoline on corneal epithelial wound healing in rat eyes. *Investigative Opthalmology & Visual Science*.

[159] Abdul-Hamid M., Moustafa N. (2014). Amelioration of alloxan-induced diabetic keratopathy by beta-carotene. *Experimental and Toxicologic Pathology*.

[160] Zagon I. S. S., ImmonenSassani J. W. W., McLaughlin P. J., McLaughlin P. J. (2014). Ocular surface abnormalities related to type 2 diabetes are reversed by the opioid antagonist naltrexone. *Clinical & Experimental Ophthalmology*.

